# Knocking out alpha-synuclein in melanoma cells dysregulates cellular iron metabolism and suppresses tumor growth

**DOI:** 10.1038/s41598-021-84443-y

**Published:** 2021-03-04

**Authors:** Sahar Shekoohi, Santhanasabapathy Rajasekaran, Dhaval Patel, Shu Yang, Wang Liu, Shile Huang, Xiuping Yu, Stephan N. Witt

**Affiliations:** Department of Biochemistry and Molecular Biology, Louisiana State University Health Sciences Center, Shreveport, LA 71103 USA

**Keywords:** Membrane proteins, Melanoma, Biochemistry, Cell biology, Molecular biology, Diseases, Oncology

## Abstract

The protein alpha-synuclein (α-syn) is unusual because, depending on its conformation and the type of cell in which it is expressed, it is pro-death or pro-survival, triggering neurodegeneration in Parkinson’s disease and enhancing cell survival of some melanomas. To probe the function of α-syn in melanoma, we used CRISPR/Cas9 to knockout *SNCA*, the gene that codes for α-syn, in SK-Mel-28 melanoma cells. The *SNCA*-knockout clones in culture exhibited a decrease in the transferrin receptor 1 (TfR1), an increase in ferritin, an increase of reactive oxygen species and proliferated slower than control cells. These *SNCA*-knockout clones grafted into SCID mice grew significantly slower than the SK-Mel-28 control cells that expressed α-syn. In the excised *SNCA*-knockout xenografts, TfR1 decreased 3.3-fold, ferritin increased 6.2-fold, the divalent metal ion transporter 1 (DMT1) increased threefold, and the iron exporter ferroportin (FPN1) decreased twofold relative to control xenografts. The excised *SNCA*-KO tumors exhibited significantly more ferric iron and TUNEL staining relative to the control melanoma xenografts. Collectively, depletion of α-syn in SK-Mel-28 cells dysregulates cellular iron metabolism, especially in xenografts, yielding melanoma cells that are deficient in TfR1 and FPN1, that accumulate ferric iron and ferritin, and that undergo apoptosis relative to control cells expressing α-syn.

## Introduction

Clinical and epidemiological studies have revealed a co-occurrence of Parkinson’s disease (PD) and malignant melanoma^1–5^. PD patients typically have a two to fourfold higher risk of developing invasive melanoma than expected in age- and sex-matched control^3^, and, reciprocally, patients with invasive melanoma have a 1.4–2-fold higher risk of developing PD^1,2^. Several genes that are associated with PD, such as alpha-synuclein (protein: α-syn; gene: *SNCA*)^6–8^, are thought to confer an increased risk of melanoma^4^. α-Syn is frequently expressed in cell lines derived from tumors representing advanced melanoma^6,9^, and tissue microarray analysis and whole-genome expression profiling show elevated levels of α-syn in advanced stage melanomas^10^. Our focus in this study was the role of α-syn in malignant melanoma.

Numerous studies have shown that α-syn plays a key role in the pathobiology of PD^11–14^. α-Syn, which is 140 amino acid protein that is expressed at quite high levels in dopaminergic neurons, but that is also expressed in a variety of other tissues, is an intrinsically unfolded protein^15^ that adopts secondary structure in a context dependent fashion. When it binds to small lipid vesicles, α-syn converts from a random coil to a bent α-helical conformation^16^. Functionally, α-syn is involved in membrane and vesicular trafficking, as it senses membrane curvature^17^, regulates synaptic vesicle fusion with the presynaptic membrane^18^, and promotes endocytosis and exocytosis^19,20^. α-Syn has a hydrophobic internal domain that can promote fibrillization, and some of the soluble amyloid fibril seeds are thought to cause neurodegeneration that defines PD^11,21,22^.

α-Syn may also have a role in iron metabolism in the brain and other tissues^23^. For example, α-syn is expressed at different steps of erythropoiesis, especially in red blood cells, and the expression of *SNCA* is significantly correlated with the expression of the heme metabolism genes *ALAS2*, *FECH*, and *BLVRB*^24^. α-Syn enhances the intake of holo-transferrin (Tf)-transferrin receptor 1 (TfR1) complexes in neuronal and non-neuronal cells, indicating that it regulates the level of iron in some cells^19,25^, and it inhibits Snx3-retromer mediated recycling of iron-regulatory proteins in a yeast PD model^26^. Brain iron deposits are also a feature of PD^27,28^.

Our interest in this study was the connection between α-syn, iron metabolism and melanoma. Because iron is used in DNA synthesis, cell-cycle progression, iron-sulfur cluster biosynthesis, heme synthesis and energy production, cancer cells have a high demand for this metal, and cancers can often subvert the normal iron homeostasis at the systemic and cellular levels to acquire high levels of iron by increasing iron uptake and storage, decreasing iron export, or both (for a review see ref^29^). At the cellular level, the two iron regulatory proteins (IREB1/2)^30^ regulate the translation of several iron metabolic proteins, including TfR1, divalent metal ion transporter 1 (DMT1)^31^, ferritin, and ferroportin (FPN1)^32^, by binding to iron-responsive elements (IREs) in the 5′ and 3′ untranslated regions of the transcripts that encode these proteins. The dysregulation of cellular iron metabolism in cancer cells can come about from mutations in oncogenes and tumor suppressors, and often these mutations cause increased cell surface expression of TfR1 or decreased cell surface expression of FPN1^29^.

Here we report that loss of α-syn expression in SK-Mel-28 cells dysregulates iron metabolic proteins, yielding cells with downregulated TfR1 and FPN1 and upregulated ferritin and DMT1. The results are consistent with loss of α-syn expression yielding melanoma cells with an overload of bio-unavailable ferric iron and a deficiency of bio-available labile iron; hence, the α-syn knockout melanoma cells, we propose, are functionally iron deficient.

## Results

### Elevated *SNCA* transcript levels correlate with poorer survival for patients with melanoma

**Figure 1:**
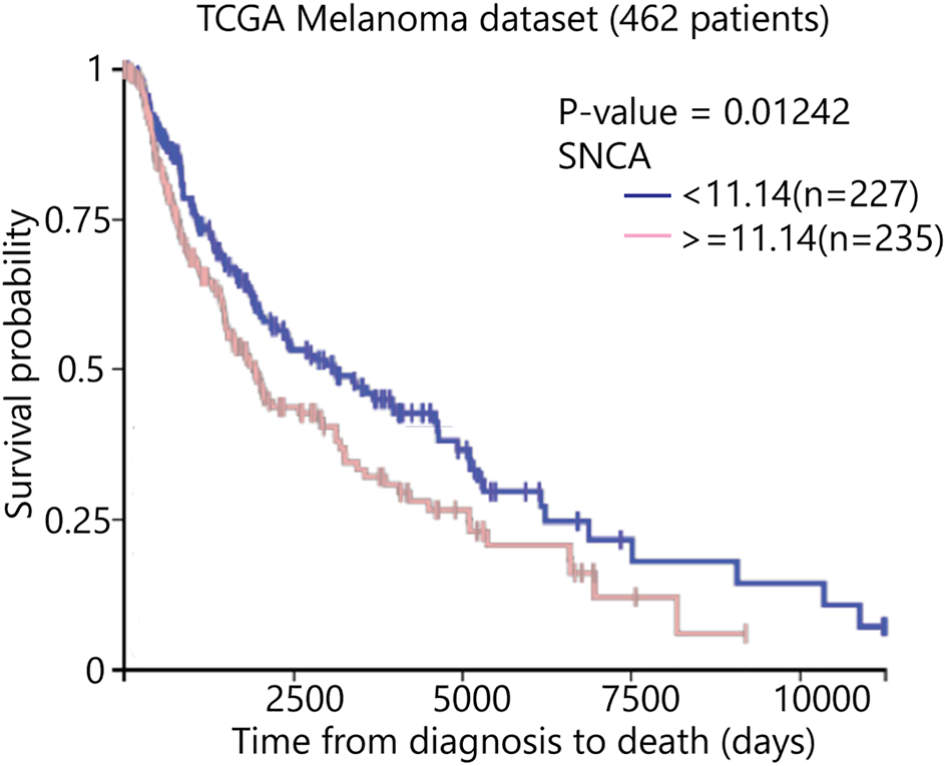
Kaplan–Meier survival curves utilizing TCGA (The Cancer Genome Atlas) melanoma mRNA expression dataset. Analysis was performed, comparing patients with high *SNCA* expression (red line) to low *SNCA* expression (blue line). Xena Browser compared the two Kaplan–Meier survival curves using the log-rank test (p = 0.01242, n = 462).

We analyzed the TGCA data set using the Xena genomics platform to determine whether elevated *SNCA* transcript levels from excised primary melanoma lesions correlate with decreased survival. The analysis of the TGCA data set (462 patients) yielded a significant association of better overall survival for patients with lower *SNCA* transcript level, while patients with high *SNCA* transcript level had worse overall survival (Fig. 1: 8.7 years [**—**] versus 5.3 years [**—**], p = 0.01242). We sought to determine herein the function of α-syn in melanoma cells.

### Loss of α-syn decreases the level of TfR1

The SK-Mel-28 melanoma cell line, which expresses α-syn^6^, is a widely used cell line^8,9,33–35^ To understand the role of α-syn in melanoma, we used CRISPR/Cas9 technology to knockout *SNCA* in SK-Mel-28 cells (Supplementary Fig. S1 and Table S1). Several of these SK-Mel-28 *SNCA*-KO clones were used herein.

**Figure 2:**
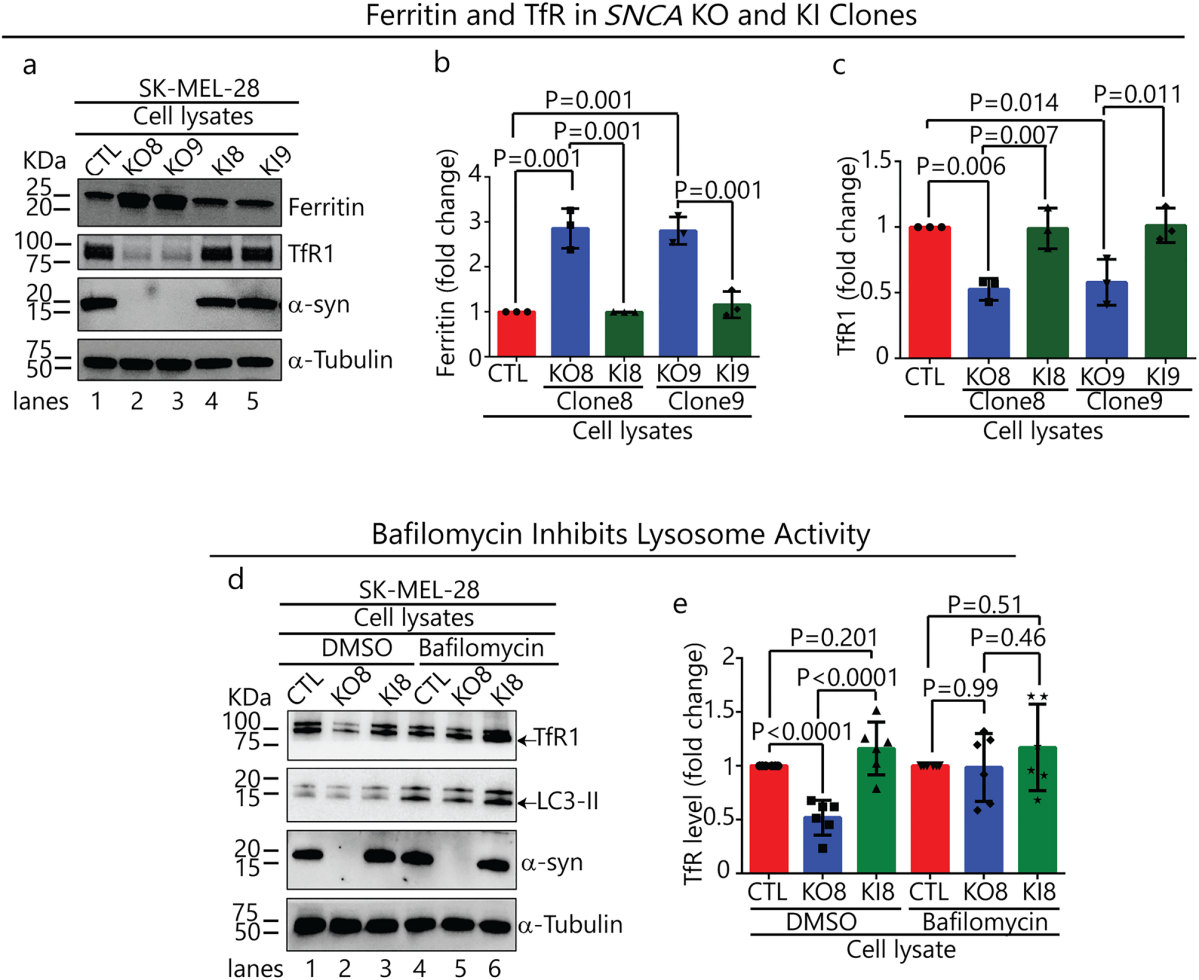
Loss of α-syn decreases the level of the TfR1 and increases the level of ferritin. (**a**) Representative Western blots of ferritin, TfR1, α-syn in lysates of the control, KO and KI cells cultured in vitro. (**b**) and (**c**) Quantitative analysis of the fold change in ferritin and TfR1. The band intensities of ferritin and TfR1 are normalized to α-tubulin. A one-way ANOVA followed by post hoc Dunnett test was used to determine p values (n = 3). (**d**) Representative Western blots showing the **e**ffect of bafilomycin A1 on the levels of TfR1, LC3-II and α-syn. Indicated cells were treated with 50 nM bafilomycin A1 for 5 h and the lysates were probed for the indicated proteins. (**e**) Quantitative analysis of the fold change in TfR1. TfR1 was normalized to α-tubulin. A one-way ANOVA followed by post hoc Dunnett test was used to determine p values (n = 6). All data are mean ± s.d. Uncropped sections of the blots are shown in Supplementary Figure S2.

TfR1 and ferritin protein levels were probed in lysates of SK-Mel-28 *SNCA*-KO clones KO8, KO9 and SK-Mel-28 control cells. To ensure that any phenotypic changes in the KO clones were due to loss of α-syn expression, α-syn was re-expressed in each KO clone using lentiviral infection, and cell lines are referred to as KI8 and KI9. Each *SNCA*-KO clone exhibited a threefold increase in ferritin (p = 0.001) (Fig. 2a, lanes 2 & 3 vs 1; Fig. 2b) and a 1.8-fold decrease in TfR1 (p = 0.014, p = 0.006) relative to control cells (Fig. 2a, lanes 2 & 3 vs 1; Fig. 2c). Strikingly, re-expressing α-syn in the clones decreased ferritin and increased TfR1 levels, such that the levels of these two proteins were indistinguishable from those in control cells (Fig. 2a, lanes 4 & 5 vs 2 & 3; Fig. 2b,c).

We asked whether the decreased level of TfR1 in the *SNCA*-KO clones was due to the degradation of TfR1 molecules in the lysosome. If TfR1 molecules are proteolytically degraded in the lysosome, then inhibiting the activity of the lysosomal proteases with bafilomycin A1 (baf) will block the degradation. To this end, cells were treated for 5 h with baf (or dimethyl-sulfoxide, DMSO, as a control) and probed for TfR1, LC3-II, α-syn and α-tubulin by Western blotting (Fig. 2d). LC3-II is a lipidated form of LC3 that builds up in autophagosomes when autophagy is inhibited by baf. In the DMSO group, the level of TfR1 decreased by 40% (p = 0.0001) relative to control cells (Fig. 2d, lanes 2 vs 1; Fig. 2e); whereas, TfR1 showed no significant decrease relative to control cells in the baf + group (Fig. 2d, lanes 5 vs 4; Fig. 2e).

**Figure 3:**
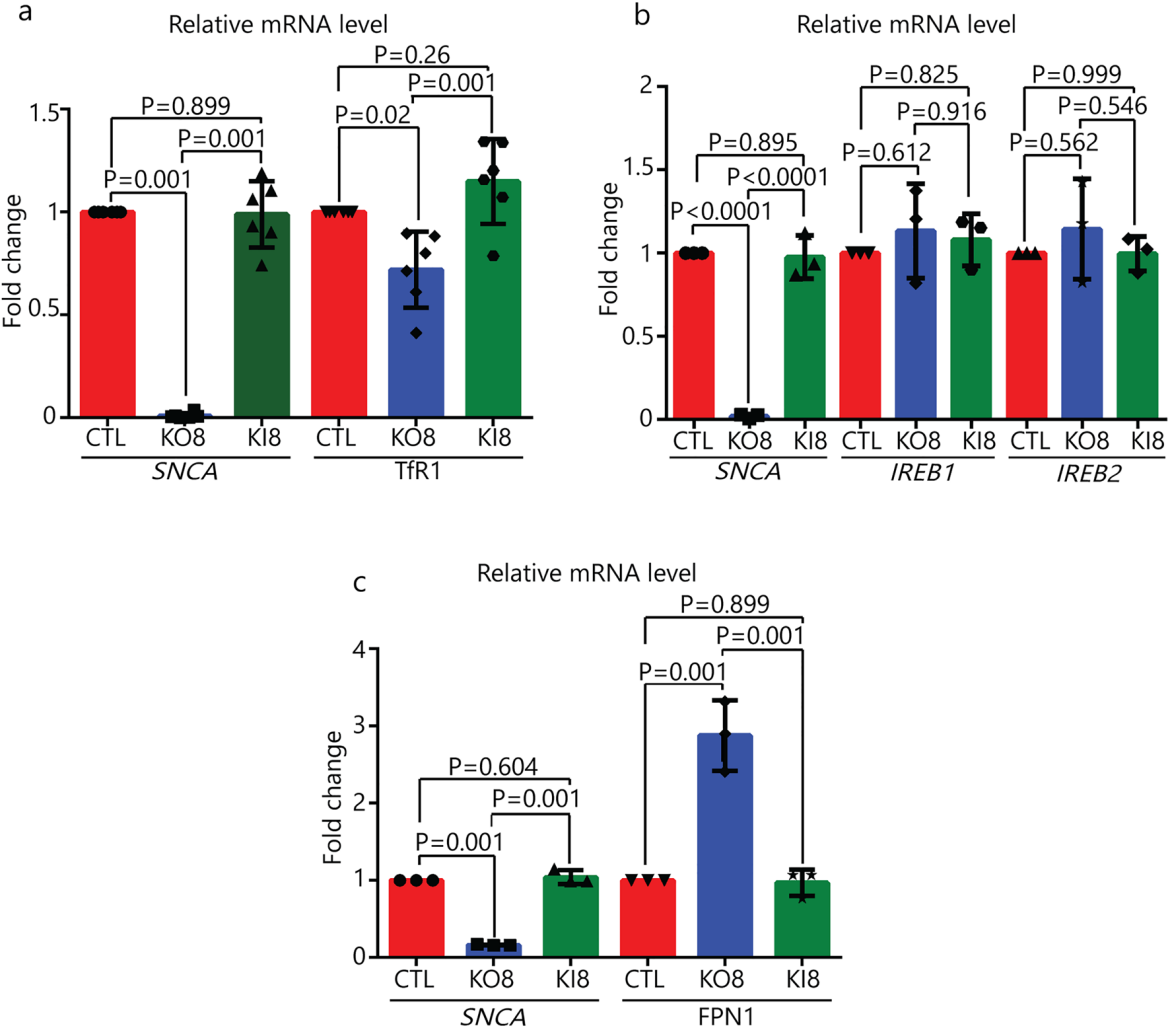
qRT-PCR analysis of iron related genes. Fold-change in mRNA level of *SNCA* and TfR1 (**a**), *SNCA* and *IREB1/2* (**b**), and *SNCA* and FPN1 (**c**) in control, KO8 and KI8 cells. Error bars are ± s.d, n = 3 per group except for TfR1 (n = 6). P-values were determined by a one-way ANOVA with Dunnet post-hoc test.

Quantitative PCR (qPCR) was also conducted to determine whether mRNA levels of several genes were affected in *SNCA*-KO clones. First, we found a modest 25% (p = 0.02), but significant, decrease in the TfR1 mRNA in KO8 cells compared to control and KI8 cells (Fig. 3a). Second, the mRNA levels of the iron responsive element binding proteins 1 and 2 (IREB1/2), which regulate the stability, and hence translation, of mRNAs of various iron-related proteins, were determined. The mRNA levels of IREB1/2 were unaffected by knocking out *SNCA* (Fig. 3b). Third, the mRNA level of the only mammalian iron exporter, FPN1, was increased by 200% in KO8 cells compared to the parental control cells, and upon re-expression of α-syn in the KO8 cells the mRNA level decreased to that of the control cells (Fig. 3c). (FPN1, see also: Dysregulation of cellular iron metabolism in *SNCA*-KO xenografts.) Overall, the data in Figs. 2 and 3 show that the decreased level of TfR1 in the *SNCA*-KO clones relative to control cells is a consequence of two phenomena: enhanced degradation in the lysosome and downregulated transcription.

### Loss of α-syn expression suppresses cell proliferation in vitro

**Figure 4:**
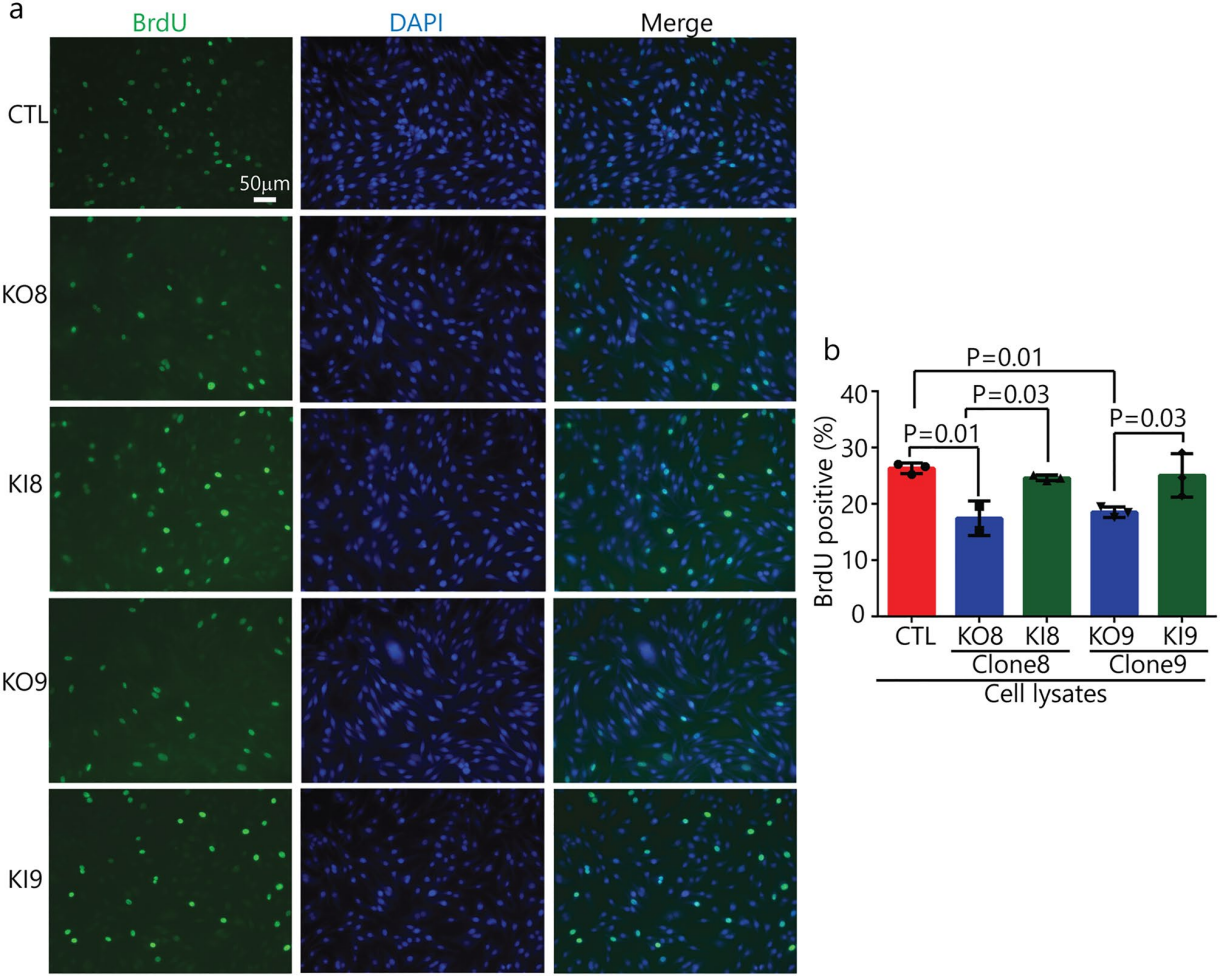
Loss of α-syn expression suppresses cell proliferation in vitro. (**a**) BrdU cell proliferation assay for control, KO and KI clones. Cells were probed with anti-BrdU antibody followed by FITC-conjugated secondary antibody (green) and counter staining the nuclei with DAPI (blue). Scale bar 50 μm magnification ×20 (n = 3). (**b**) Quantitative data expressing BrdU positive cells (in green) was evaluated by counting the positively stained cells in a blinded fashion by an unbiased observer utilizing Image J software. The average number of BrdU positive cells across 3 fields/slide was used to denote the total number of positively stained cells and expressed as percentage of BrdU positive cells. A one-way ANOVA with Dunnett test was performed to calculate p values.

BrdU is an analog of thymidine, which can be incorporated into the newly synthesized DNA in replicating cells; thus, BrdU labeling is commonly used to detect DNA synthesis or cell proliferation^36^. A BrdU incorporation assay was used to determine whether knocking out α-syn affects proliferation in vitro. KO8 and KO9 clones and the corresponding KI clones were tested against the control SK-Mel-28 cells. The percentage of BrdU + cells was determined by fluorescence microscopy analysis, using DAPI to determine the number of total cells per field. On average, there was a significant (p = 0.01) decrease in BrdU incorporation in the two KO clones compared to SK-Mel-28 control cells, and the KI clones had BrdU incorporation values similar to the control cells (Fig. 4a,b).

### Loss of α-syn expression suppresses melanoma tumor growth in a mouse xenograft model

**Figure 5:**
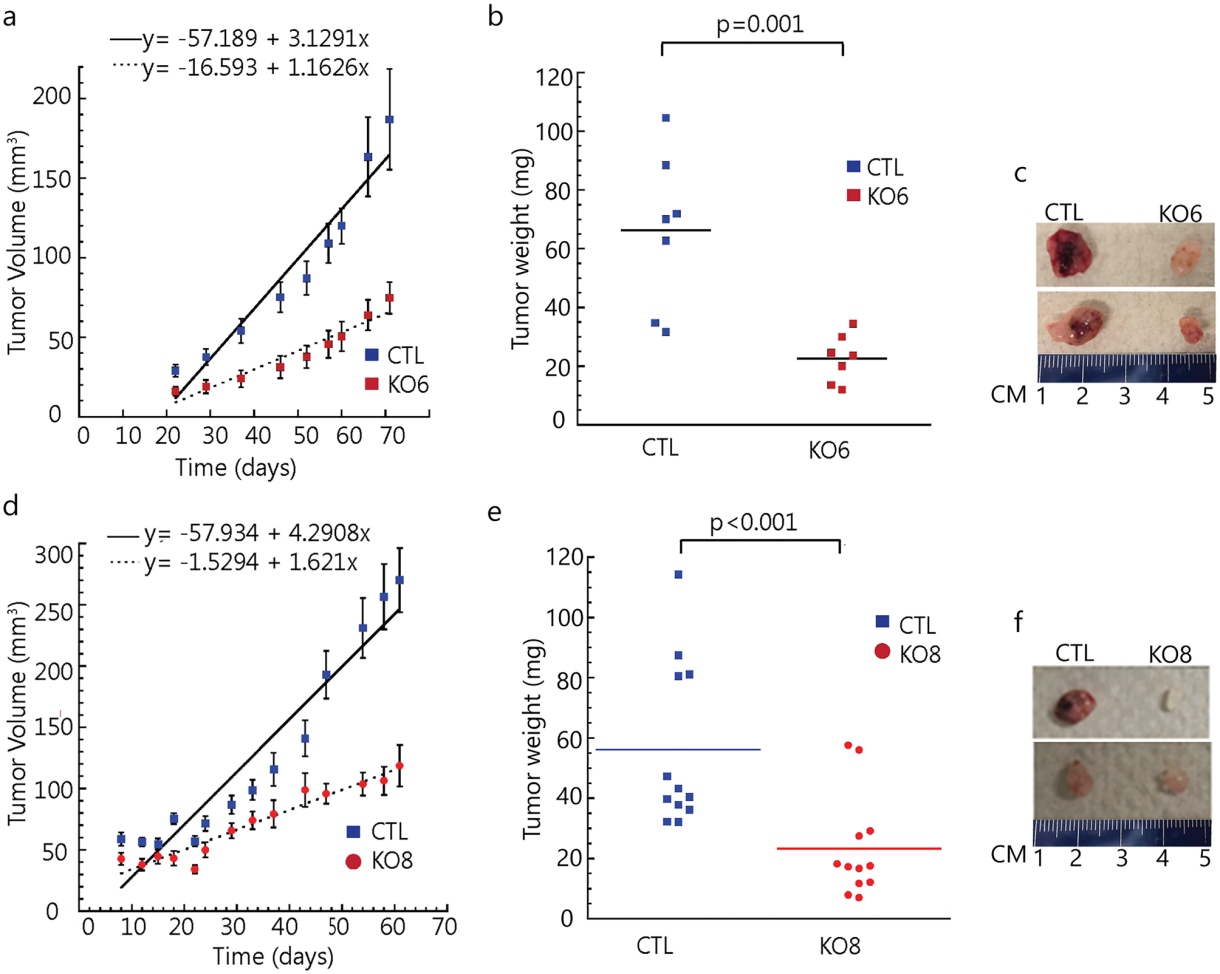
Loss of α-syn expression suppresses cell growth in a mouse xenograft model. (**a**) and (**d**) Tumor volume over time for the control, *SNCA,* KO6 and KO8 mice xenografts. Tumor volume was assessed every 2 days, and average tumor weight was determined after the mice were sacrificed at the end of 72-day experiment. (**b**) and (**e**) Weight of the excised tumors are shown. (**c**) and (**f**) Representative photographs of xenograft mice tumors. Tumor volume and weight were analyzed using two-tailed Student's t test (n = 7 for KO6 and n = 12 for KO8).

**Figure 6:**
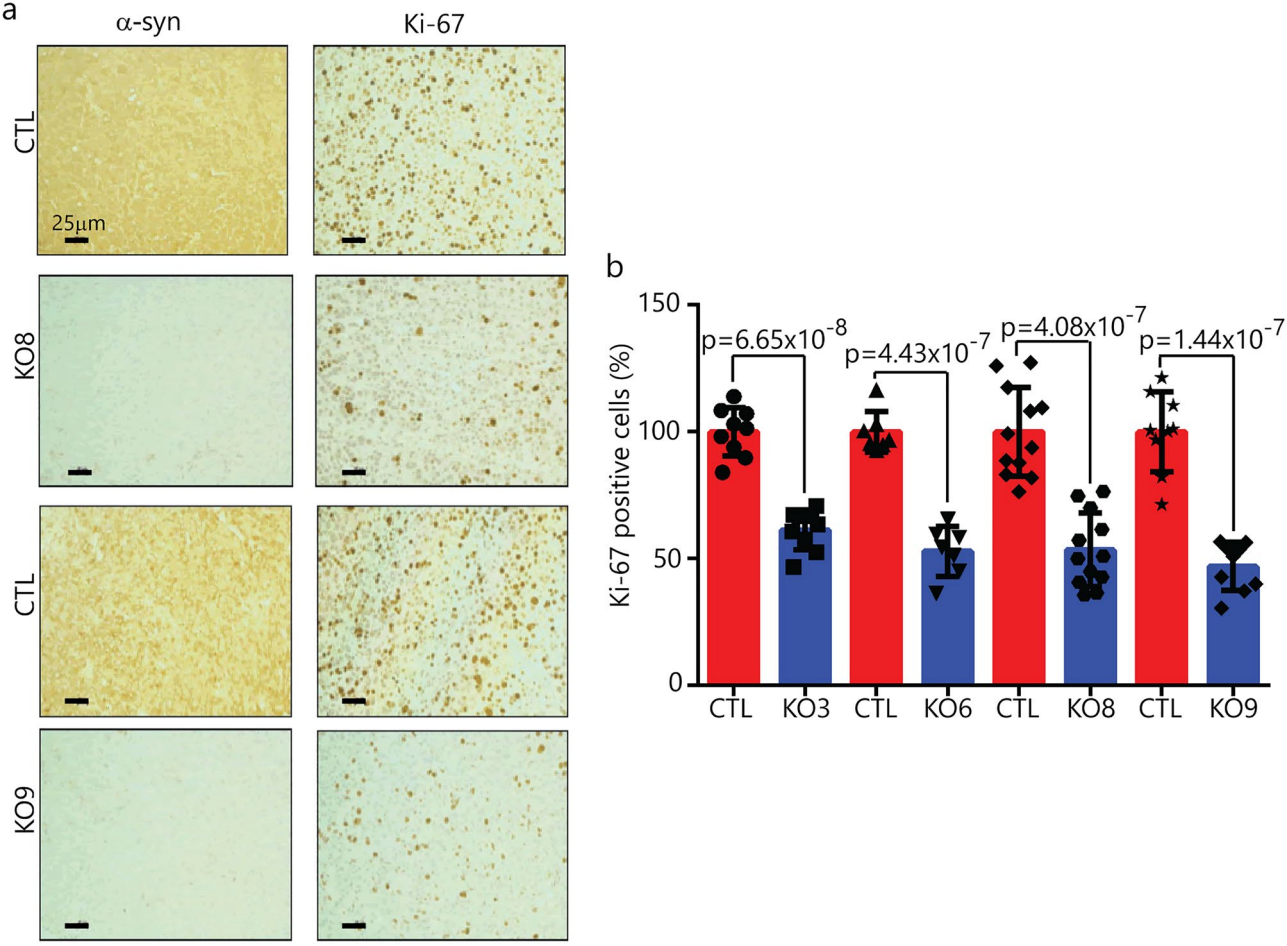
α-Syn and Ki-67 staining of tumor tissue from SK-Mel-28 xenografts. (**a**) Representative images of immunostained sections of xenografts generated from subcutaneous injection of the *SNCA* KO clone 8, 9 and control cells into mice. Tumor sections were probed with antibodies specific for α-syn and Ki-67. Magnification 20 × , scale bar = 25 μm. (**b**) Plot of the number of Ki-67 immunopositive cells per ×20 field. For each condition, n = 3 (KO3, 6, 9) and n = 4 (KO8) xenografts, with 1–3 fields counted for each slide. The two tailed student's t test was performed to calculate p values. Data for clones KO3 and 6 are given in the Supplementary section. Error bars are ± s.d.

We sought to determine whether the finding of suppressed proliferation in vitro translates to an in vivo model. To that end, in the same SCID mouse, we injected 1 × 10^6^ SK-Mel-28 control cells in matrigel subcutaneously into the left flank in mice, and the same number of SK-Mel-28 *SNCA*-KO cells in matrigel were injected subcutaneously into the right flank. Clones KO3, KO6, KO8 and KO9 were tested. The animals were followed for 72 days and then sacrificed. Tumor volume curves for clones 6 and 8 are shown in Fig. 5a,d, respectively. Tumor volume data for KO3 and KO9 are given in Supplementary Fig. S3. The two KO clones grew much slower than the control cells (Fig. 5a,d), and, at the endpoint, average weight of the tumor formed from the KO clones was significantly (p = 0.001) less than the control tumors (Fig. 5b,c,e,f). Tumor sections were also evaluated by IHC to probe for α-syn and Ki-67 (Fig. 6), which is a nuclear protein associated with cell proliferation. KI-67 immunostaining is frequently used to evaluate cell proliferation in tissues^37^. For each KO clone tested, Ki-67 decreased significantly compared to the control tumor (Fig. 6a,b; Supplementary Fig. S4). IHC also showed complete lack of staining for α-syn in the KO clones. Overall, the data show that knocking out α-syn in melanoma cells suppresses the tumor growth at least partly by inhibiting tumor cell proliferation.

### Dysregulation of cellular iron metabolism in *SNCA*-KO xenografts

**Figure 7:**
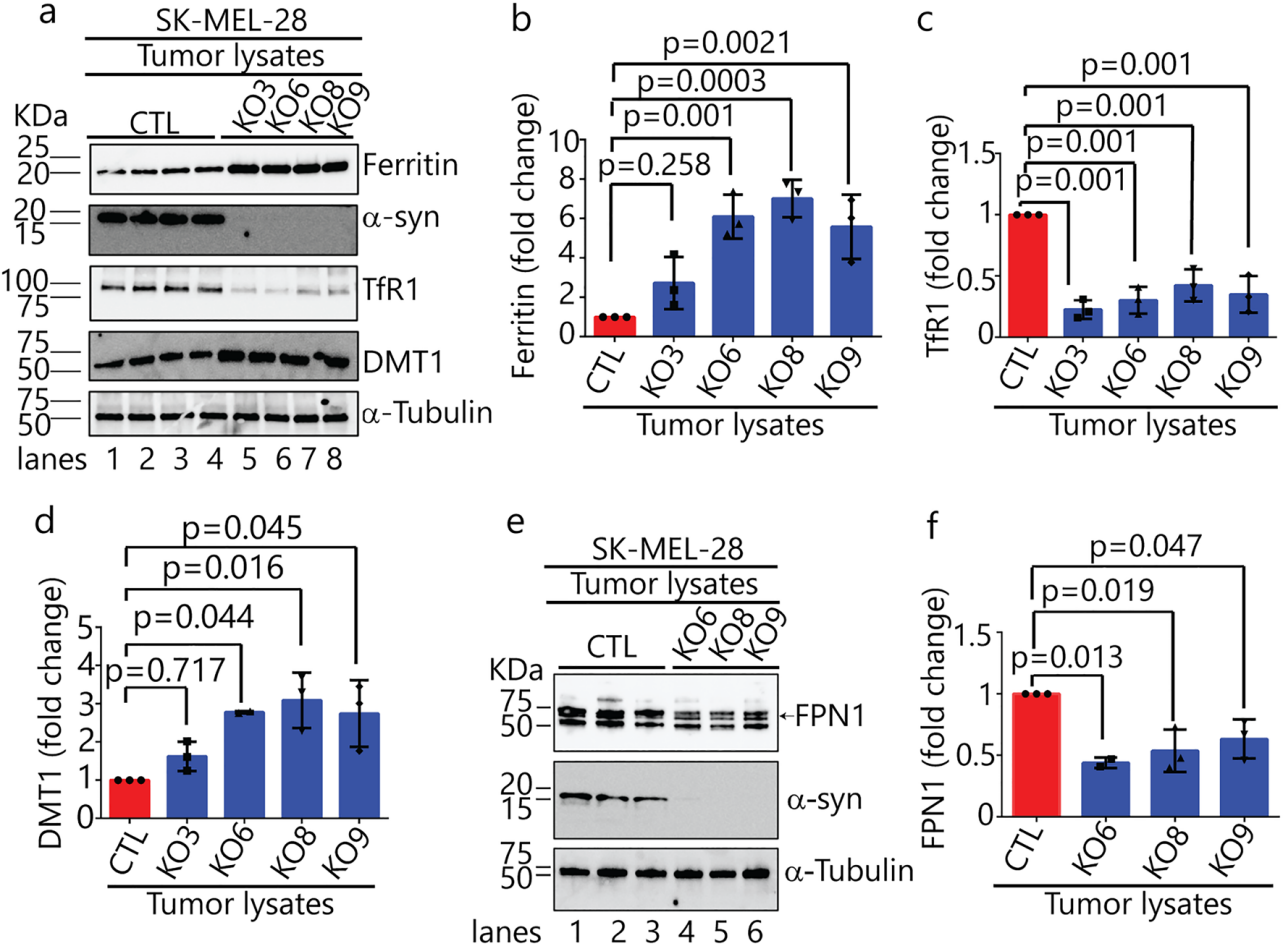
Loss of α-syn alters levels of proteins involved in iron-homeostasis. (**a**) Representative Western blots of iron-related proteins (ferritin, α-syn, TfR1, and DMT1) in the indicated xenograft lysates. (**b**–**d)** Quantitative analysis of fold changes in ferritin, TfR1 and DMT1, derived from densitometric analysis of band intensities normalized to α-tubulin. P-values were determined using a one-way ANOVA with Dunnett posthoc test (n = 3). (**e)** Representative Western blots of ferroportin (FPN1) and α-syn in the indicated xenograft lysates. (**f**) Quantitative analysis of the fold change in FPN1, derived from densitometric analysis of band intensities normalized to α-tubulin. P-values were determined using a one-way ANOVA with Dunnett posthoc test (n = 3). Error bars in each plot are ± s.d. Uncropped sections of blots are shown in Supplementary Figure S5.

Lysates of tumors excised from the SCID mice were western blotted to quantify the levels of ferritin, TfR1, DMT1 and FPN1. DMT1 isoform 2 localizes to endosomes, where it is activated at pH 5.5 to pump iron (in the ferrous state) from the lumen of endosomes into the cytosol^38^. FPN1 localizes to the plasma membrane, where it pumps iron (in the ferrous form)^39^ out of cells. Statistically significant changes in protein levels were identified in *SNCA*-KO xenografts, compared to controls, as follows: (i) ferritin increased 6.2 (± 0.5)-fold in xenografts 6, 8 and 9 (Fig. 7a,b); (ii) TfR1 decreased 3.3 (± 7)-fold in all four xenografts (Fig. 7a,c); (iii) DMT1 increased 2.9 (± 0.1)-fold in xenografts 6, 8 and 9 (Fig. 7a,d); and (iv) FPN1 decreased 1.9 (± 0.3)-fold in xenografts 6, 8 and 9 (Fig. 7e,f). Collectively, loss of α-syn affects the levels of both iron import (TfR1/DNM1) and iron export (FPN1) proteins. Moreover, because ferritin levels were significantly increased in the *SNCA*-KO clones compared to control cells, we hypothesized that knocking out α-syn dysregulates iron metabolism in such as manner as increase the concentration of non-labile, ferric iron.

### Increased ferric iron in *SNCA*-KO xenografts

**Figure 8:**
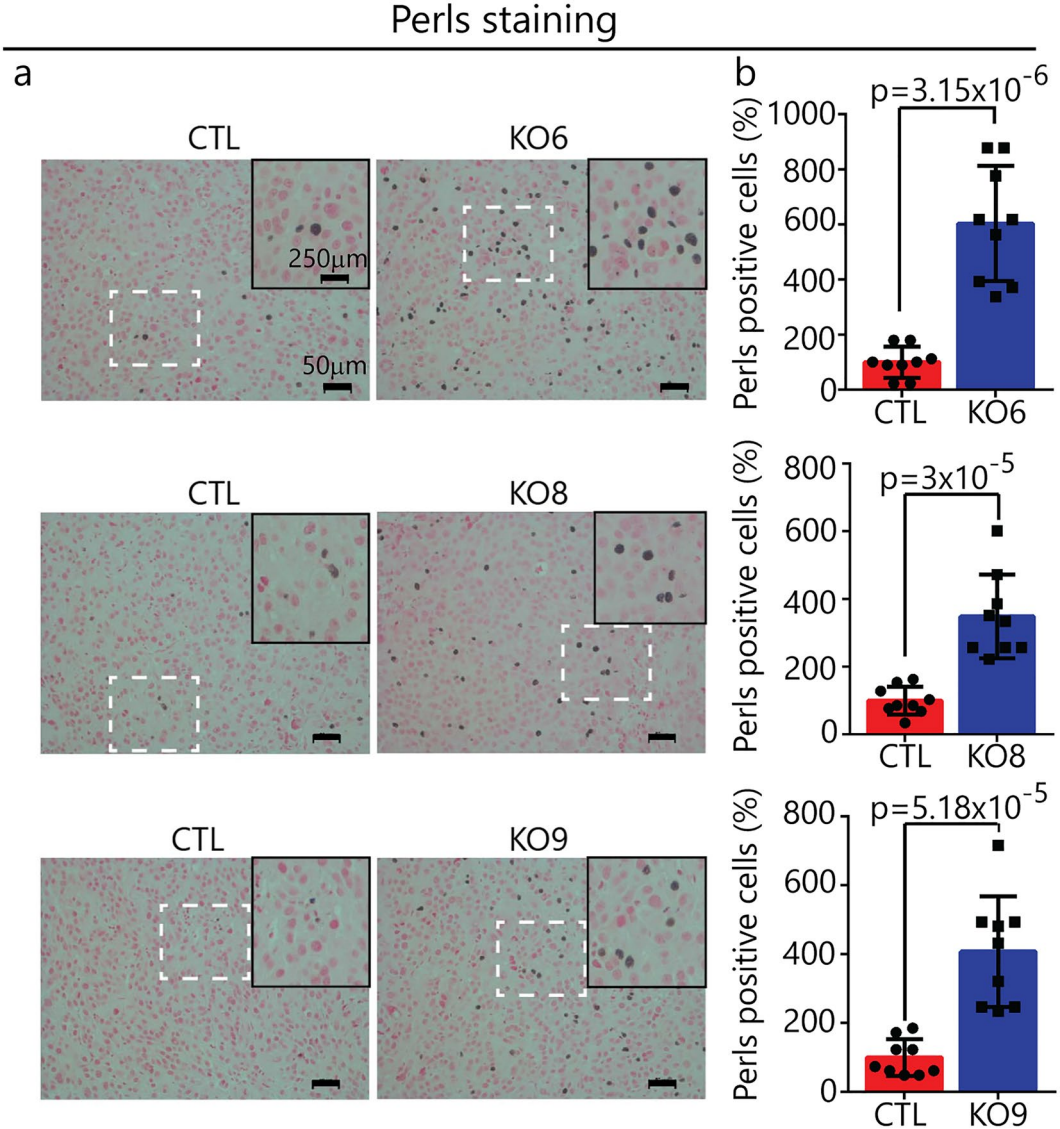
Iron accumulation in *SNCA* KO xenografts. (**a**) Representative images of Pearl’s stained xenograft tissue sections. Inner panel shows zoomed in region of interest from each section for better illustration. Histological sections stained with Perls Prussian blue and Nuclear fast red indicate the presence of hemosiderin (blue-black stain). Iron in hemosiderin (an iron-storage complex) turns blue to black when exposed to potassium ferrocyanide (Prussian blue stain). Magnification: ×20, Scale bar: 50 μm. (**b**) Plots of the average number of black puncta (hemosiderin) per unit area (×20 field) for control and KO xenografts. For each condition, n = 3 xenografts, with 3 fields counted for each slide (= 9 fields). A two tailed student's t test was used to calculate p values. Error bars are ± s.d.

To test the hypothesis that loss of α-syn expression in SK-Mel-28 cells dysregulates the iron import–export balance towards ferric iron accumulation, excised xenograft sections were analyzed by Perls staining, which is a technique used to detect non-heme ferric iron (Fe^3+^), such as occurs in ferritin and hemosiderin, in tissue sections^40^. After treatment of the tissue with ferrocyanide and acid, deposits of iron appear as brown puncta. Puncta area readily visualized by light microcopy and the number of puncta per unit area quantified. Applying this technique to clones KO6, 8 and 9 and SK-Mel-28 control xenografts, we found significantly (p = 0.0001) more ferric iron deposits in each of the KO xenografts compared to control cells (Fig. 8a,b). Ferric iron deposits per unit area were typically three to fivefold higher than the density of iron deposits in the control xenografts.

### Increased apoptosis in *SNCA*-KO xenografts

**Figure 9:**
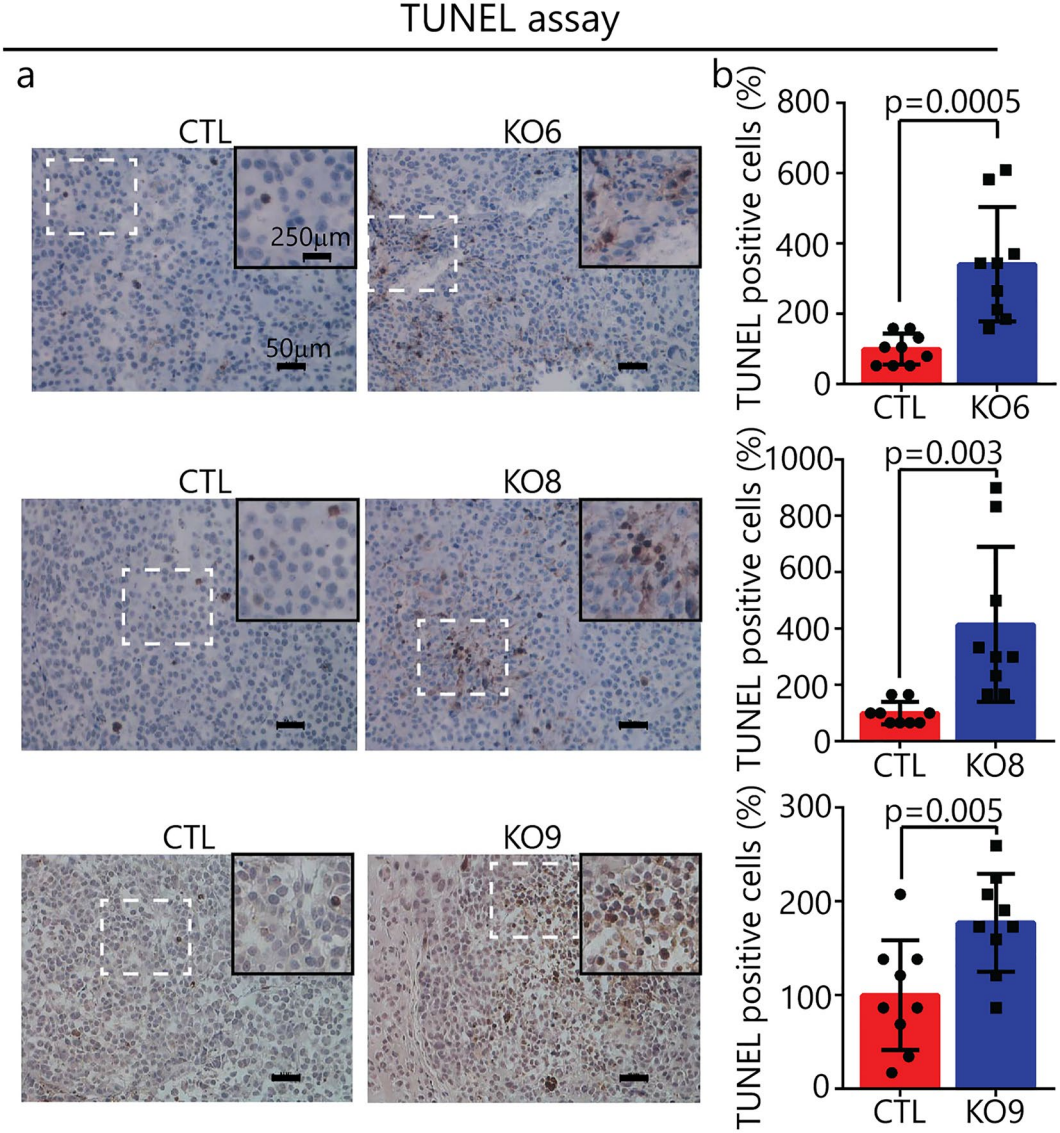
Apoptosis in in *SNCA* KO xenografts. (**a**) Representative xenograft tissue sections from control and KO tumors following the TUNEL assay. Magnification: ×20, Scale bar: 50 μm. (**b**) Plots of average number of TUNEL positive cells per unit area (×20 field) in control and KOs. For each condition, n = 3 xenografts, with 3 fields counted for each slide (= 9 fields). A two tailed student's t test was used to calculate p values. Error bars are ± s.d.

Apoptosis can be caused by iron deficit or iron overload^41–43^. Given that *SNCA*-KO xenografts exhibited increased levels of both ferric iron and ferritin relative to controls, we asked whether these xenografts also exhibited increased apoptosis. To that end, xenograft sections were analyzed for DNA fragmentation using the TdT-mediated, dUTP-biotin nick-end labeling (TUNEL) assay. The TUNEL assay is a method for detecting DNA fragmentation in cells, and it is frequently used to evaluate apoptosis in tissues^37^. TUNEL analysis of xenografts from KO6, 8 and 9 and controls revealed that the three KO clones showed significantly more DNA fragmentation per field than the controls (Fig. 9a,b). On average, the sections from the KO xenografts had two to threefold higher amounts of DNA fragmentation that the controls. Collectively, *SNCA*-KO xenografts had higher levels of both ferric iron and apoptotic TUNEL markers than the control xenografts (Figs. 8, 9).

## Discussion

In this study, we engineered SK-Mel-28 α-syn knockout cells (SK-Mel-28 *SNCA*-KO) to investigate the role, if any, played by α-syn protein in promoting proliferation (Fig. 1). Our results are consistent with the loss of *SNCA* significantly suppressing tumor growth, and the suppressed tumor growth is a consequence of a disturbance in iron metabolism. Aspects of this work are discussed below.

The synuclein family contains three (α, β, and γ-synuclein) highly conserved, small, soluble membrane-binding proteins^44^. α-Syn regulates membrane trafficking events ^20,45^, whereas much less is known about the function of β- and γ-syn. These proteins are highly expressed in brain and to a lesser extent in other tissues^46^. While α-syn has the greatest propensity to form soluble and insoluble neurotoxic aggregates and amyloid fibers that cause neurodegeneration, aggregated forms of β- and γ-syn may also contribute to some neurodegenerative diseases^47,48^. There is an intriguing connection of synucleins to cancer^5,49^, which suggests that neurodegeneration and cancer may share common mechanisms: (i) α-syn is highly expressed in melanoma cell lines^6^, and over-expressing α-syn promotes the proliferation of B16 murine melanoma cells^50^ as well as human SH-SY5Y neuroblastoma cells^51^. The mechanism by which α-syn promotes proliferation is unclear. (ii) β-syn is over-expressed in breast cancers^52^. (iii) γ-syn is abnormally expressed in various malignancies, but normal tissue, including breast, cervical, colon, prostate and ovarian cancers^52,53^. γ-syn promotes cancer cell migration and invasion^54^, and down regulating γ-syn expression by siRNA decreases cervical cancer proliferation in nude mice^55^. The role of synucleins in cancer is wide-open area of research.

**Figure 10:**
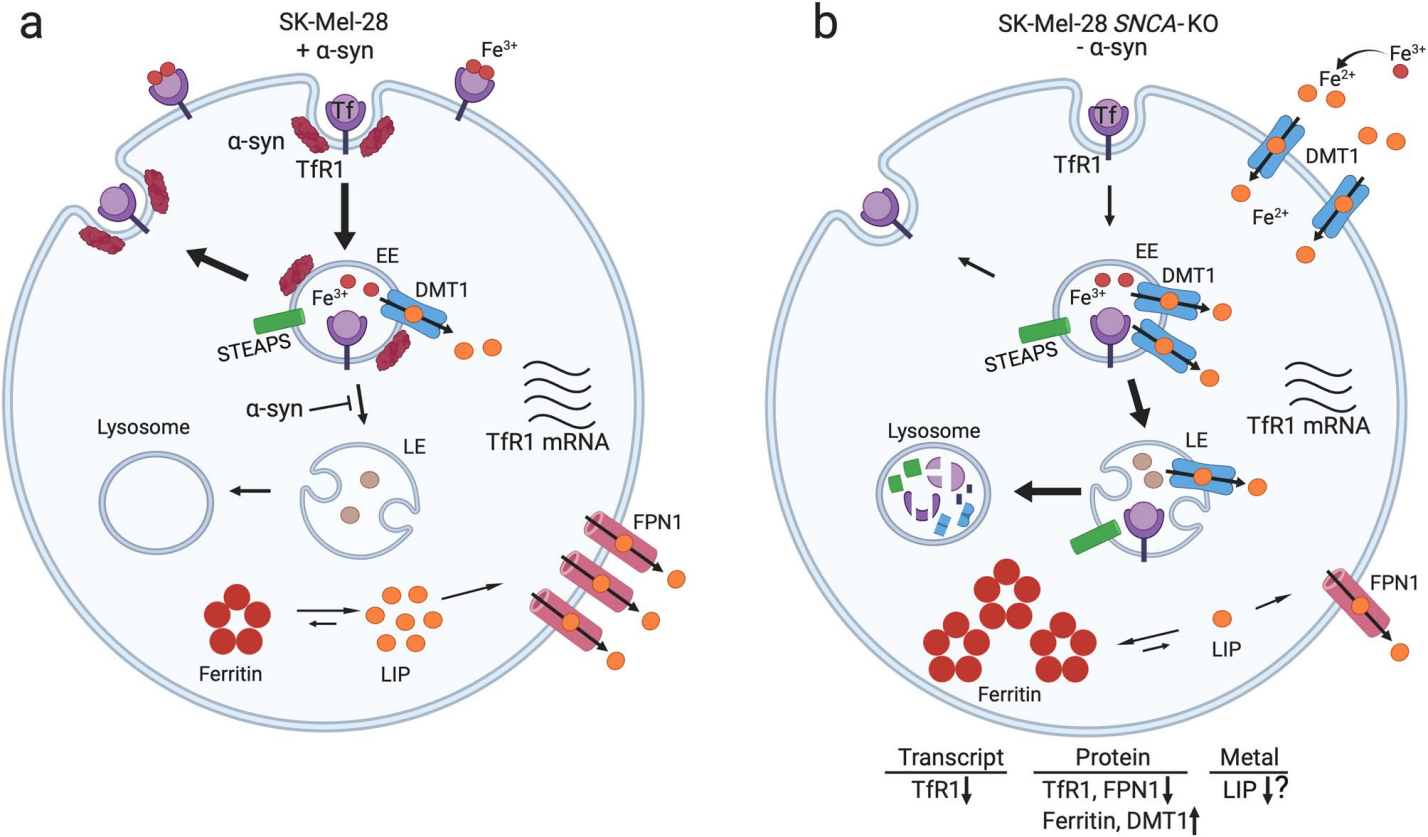
Model for the dysregulation of iron metabolic proteins in *SNCA*-KO melanoma cells. (**a**) To acquire iron for the enzymes of respiration, DNA synthesis and repair and cluster iron-sulphur production, cancer cells often exhibit an “iron-seeking phenotype,” i.e., increased levels of transferrin receptor 1 (TFR1), six-transmembrane epithelial antigen of prostate (STEAP) proteins, divalent metal ion transporter 1 (DMT1), and hepcidin (HP) (not shown here) and decreased level of ferroportin (FPN1) compared with normal cells of the same tissue^29^. (**b**) SK-Mel-28 *SNCA*-KO melanoma clones exhibit an increase in ferric iron and ferritin and a decrease in TfR1 receptors relative to control cells. DMT1 protein level is increased relative to control cells, and FPN1 is transcriptionally upregulated (Fig. 3) although its expression is lower than in control cells (Fig. 7e,f). *SNCA*-KO melanoma cells are overloaded with ferritin-ferric iron and likely have a low level of useable ferrous iron (labile iron pool, LIP) relative to SK-Mel-28 control cells. Such *SNCA*-KO cells are functionally iron deficient. The question mark (?) indicates that we have not proved that LIP is low. The red arrows show the direction of change relative to the SK-Mel-28 control cells.

We found that the SK-Mel-28 *SNCA*-KO cells in culture or implanted in mice exhibit significantly lower levels of TfR1 relative to control cells (Figs. 2a,c, 7a,c). In the mouse model, the SK-Mel-28 *SNCA*-KO melanoma xenografts compensate for the relatively low level of TfR1, which should cause iron deficiency, by decreasing the level of FPN1 (Fig. 7e,f), which decreases the efflux of iron out of cells, and increasing DMT1 (Fig. 7a,d), which, promotes ferrous iron export from endosomes into the cytosol (Fig. 10). We do not know why ferritin increases so dramatically in the *SNCA*-KO melanoma xenografts; nevertheless, the KO xenografts accumulate large amounts of ferritin/ferric iron, indicating a misregulation of iron inside the tumors. Our interpretation is that the *SNCA*-KO melanoma xenografts are overloaded with bio unavailable ferric iron bound to ferritin, and such cells, we speculate, probably have low amounts of bioavailable ferrous iron; hence, *SNCA*-KO melanoma cells are likely to be functionally iron deficient (Fig. 10). A functional iron deficiency, which could cause defects in DNA synthesis, cell cycle progression and energy production, likely causes the slow growth phenotype when the SK-Mel-28 *SNCA*-KO melanoma clones are engrafted into SCID mice (Figs. 5, S3). In support of our interpretation, when SK-Mel-28 cells are engrafted into SCID mice and the mice are treated with the iron chelator Dp44mT the chelator dramatically inhibits tumor growth^33^, which shows that depleting iron from melanoma cells inhibits their growth and proliferation.

FPN1 is transcriptionally upregulated in the SK-Mel-28 *SNCA*-KO clones in culture compared to control cells (Fig. 3c). Given that *SNCA*-KO xenografts accumulate ferric iron (Fig. 8), in our view, it is not surprising that cells overloaded with iron respond by upregulating the expression of an iron exporter. Curiously, however, the level of the FPN1 protein is decreased in *SNCA*-KO xenografts relative to the control xenografts. This decrease in FPN1 is most likely due to enhanced degradation of FPN1 in the lysosome. We suggest that both membrane proteins, TfR1 and FPN1, which cycle between the plasma membrane and locations within the cell, such as the trans-Golgi, are degraded faster in the *SNCA*-KO cells than in control cells. Overall, loss of α-syn expression accelerates the lysosomal degradation of TfR1, and likely FPN1.

Other studies have also shown that α-syn promotes the uptake of transferrin-bound iron (Tf-Fe^3+^)^19,25^. First, one study explored the effect of α-syn expression on the endocytosis of the fluorescent dye FM-464 on a neuronal cell line in cultural. α-Syn and physiological concentrations of certain polyunsaturated fatty acids (PUFAs) were shown to act synergistically to enhance the endocytosis of FM-464^19^. Additionally, α-syn alone, PUFA alone, or PUFA+  α-syn in an additive manner enhance clathrin-mediated endocytosis of a fluorescently tagged transferrin (Alex-488 Tf) in neuronal and non-neuronal cultured cells^19^. Overexpression of α-syn in MN9D neuronal cells caused a threefold increase in the TfR1 mRNA compared to control cells with no α-syn expression. Second, retinal epithelial (RPE) cells express α-syn, and Parkinson’s patients often experience ocular problems. Knocking down α-syn expression in retinal epithelial (RPE) cells significantly decreases the levels of both TfR1 and ferritin compared to control RPE cells^25^; whereas, overexpressing α-syn significantly increased the levels of these two proteins. Furthermore, knock down of α-syn appeared to shift TfR1 molecules into perinuclear vesicles, suggesting disrupted TfR1 recycling to the plasma membrane, and such vesicles are likely degraded in the lysosome^25^. We have shown that a subpopulation of TfR1 molecules are also degraded in the lysosome in SK-Mel-28 *SNCA*-KO melanoma cells (Fig. 2d,e).

Iron metabolism appears to be disrupted quite differently in the SK-Mel-28-*SNCA*-KO cells and homozygous *SNC*A-KO RPE cells. For example, loss of α-syn expression in the SK-Mel-28 cells yields a decrease in the level of TfR1 and an increase in the level of ferritin, generating a ferric iron overload. In contrast, loss of α-syn expression in RPE cells yields a decrease in levels of both TfR and ferritin, in this case, generating a ferric iron deficiency. These disparate results indicate that very different regulatory mechanisms are at work in these two types of cells.

In sum, knocking out α-syn expression in SK-Mel-28 cells has two effects on TfR1: the TfR1 mRNA is slightly but significantly decreased and degradation of TfR1 in the lysosome is enhanced relative to control cells that express α-syn. Whether TfR1 molecules are stuck on the plasma membrane due to a defect in endocytosis or stuck in Golgi vesicles due to a trafficking defect will be determined in future experiments. Overall, loss of α-syn in SK-Mel-28 cells dysregulates cellular iron metabolic proteins, leading to ferritin-ferric iron accumulation, and apoptosis.

## Materials and methods

### Animal maintenance and xenografts

All animals in this study received humane care in accordance with the guidelines set by the American Veterinary Medical Association, and all experiments were approved by the Institutional Animal Care and Use Committee of LSU Health Sciences Center in Shreveport (LSUHSC-S IACUC). Protocol #P-18-019. Female SCID-Beige mice (Taconic) were used at 7–8 weeks of age. Mice were maintained at LSU Health Sciences Center-Shreveport animal facility in sterile cages. Groups KO3, KO6, KO8, and KO9 contained 9, 7, 12, and 9 mice, respectively. To create xenografts, 1 × 10^6^ *SNCA*-KO clones and SK-Mel-28 control cells, suspended in 50 µl of growth medium and mixed with 50 µl of matrigel (Corning #354234), were injected subcutaneously into the left and right back (2 grafts per each mouse) in SCID mice. Tumors were measured with digital calipers (3 times/week) and tumor volume calculated as: tumor volume (mm^3^) = (length × width^2^) × 0.5236^56^. Mice were sacrificed by isoflurane overdose and death was confirmed by cervical dislocation.

### Antibodies and reagents

α-Syn (610786) was purchased from BD Biosciences, transferrin receptor (13-6800) and ferroportin (PA522993) from Thermo Fisher Scientific, LC3-II (2775S) from Cell Signaling Technology, α-tubulin (T9026), HRP-conjugated anti-rabbit and anti-mouse antibodies (sc-516102, sc-2357), NRAMP (sc-166884), ferritin (sc-74513). α-Syn CRISPR/Cas9 knockout plasmid (sc-417273) from Santa Cruz Biotechnology. Bafilomycin A1 (B1793-10), 2′,7′-dichlorofluorescin diacetate (D6883) and DMSO (D8418) were obtained from Sigma. Penicillin–streptomycin (15140122) and phosphate-buffered saline (PBS-10010031) were from Gibco. Dulbecco's Modified Eagle's Medium (DMEM) (63844117), trypsin/EDTA (30-2101) and fetal bovine serum (30-2020) were purchased from American Type Culture Collection (ATCC, Manassas, VA).

### Cell line and cell culture

The SK-MEL-28 cell line, which was purchased from ATCC, were authenticated at the University of Arizona Genetics Core via their STR Profiling Cell Authentication Service. Cells were cultured in DMEM supplemented with 10% fetal bovine serum (FBS) and 1% penicillin–streptomycin. The SK-MEL-28 cell line, and all knockout (KO) and knockin (KI) cell lines, were maintained in a humidified chamber with constant supply of 5% CO_2_ and 95% O_2_ at 37 °C.

### CRISPR/Cas9-mediated gene knockout

CRISPR/Cas9 genome editing was used to target *SNCA* in SK-MEL-28 cells, α-syn CRISPR/Cas9 knockout plasmid containing 3 guide RNAs and GFP (as a marker for transfection) was purchased from Santa Cruz Biotechnology. Guide-RNA 1, 2 and 3 were complementary to exon 3, 4 and 5, respectively. Therefore, the Cas9 cut exon 3 and/or 4 and/or 5. Single cells expressing GFP were sorted in 96 well plates by fluorescence activated cell sorting. Further, we performed expansion of clones from single cells to 24 well, 12 well, 6 well plates, T25 and T75 flasks followed by validation of the *SNCA* knockout clones.

### Re-expression of α-syn in *SNCA* KO clones

Lentivirus particles (> 10^7^ IU/ml) expressing human α-syn under CMV promoter (pLenti-GIII-CMV-GFP-2A-Puro) were procured from Applied Biological Materials. Inc, Canada. *SNCA* KO cells were transduced at a multiplicity of infection 1 in a fresh DMEM with 10% (v/v) FBS and 8 µg/mL of polybrene (Sigma, St Louis, MO, USA) for 6 h. After 48 h the cells were selected with puromycin (0.5 µg/ml) for 7 days. The positively selected surviving cells were then sorted by FACS followed by expansion in 96 well plates, moved to 24 well, 12 well, 6 well plates, T25 flask and screened for the presence of α-syn by immunoblotting analysis.

### RNA extraction, cDNA preparation and qPCR

Cells were harvested and total RNA of cells was extracted from *SNCA* knockout clones and control cells using E.Z.N.A column-based total RNA kit (Omega BioTek). Total RNA concentration was determined using Nanodrop spectrophotometer and 1 μg total RNA was used to prepare cDNA following the manufacturer’s protocol. cDNA was synthesized from total purified RNA by using iScript cDNA synthesis kit (Bio-Rad) according to the manufacturer’s protocol. qPCR reactions were performed using SYBR green PCR super mix reagent (Bio-Rad). β-actin was used as a housekeeping gene to normalize Ct values. The relative expression of each amplicon was analyzed by the delta Ct method. Primer sequences were *SNCA* (Forward: 5′-ACTGCTCCTCCAACATTTGTC-3′ and Reverse: 5′-AGGGTGTTCTCTATGTAGGCT-3′) and β-Actin (Forward: 5′-GGCATCCTCACCCTGAAGTA-3′ and Reverse: 5′-CAGAGGCGTACAGGGATAGC-3′). TaqMan primers for qPCR were *SNCA* (Hs00240906), *TfR1* (Hs00951083-m1), *IREB1* (Hs00158095-m1), *IERB2* (Hs01021795-g1) and *FPN1* (Hs00205888-m1) (ThermoFisher).

### Western blotting

Cells were lysed in RIPA lysis buffer (50 mM Tris HCL, pH 7.4, 1% NP-40, 0.5% sodium deoxycholate, 0.1% SDS, 5 mM EDTA) followed by centrifugation (13,000 rpm/30 min/4 °C). the protein concentrations of the supernatants were determined using DC Protein Assay Kit (BIO-RAD 5000112), and all other steps performed as described^26,57^. Given that we typically detected multiple proteins from a single immunoblot, membranes were often cut into strips and the individual strips were hybridized with antibody.

### Immunohistochemistry

Paraffin embedded tissue sections of 5 μm thickness were deparaffinized in xylene and then rehydrated in graded alcohol series (100%, 95%, 70%, 50%) solutions followed by heating in a microwave (20 min) for antigen retrieval using antigen unmasking solution (Vector Laboratories, H-3300-250). To inhibit endogenous peroxidases, sections were incubated with 0.3% H_2_O_2_ in methanol at room temperature and then cleared in PBS for 5 min. Slides were incubated in blocking solution (5% either horse (Vector Laboratories, s-2000) or goat (s-1000) serum in 1XPBS) for 30 min at room temperature. The sections were incubated overnight with mouse monoclonal α-synuclein antibody (1:500) and rabbit monoclonal Ki-67 antibody (1:500) in blocking solution at 4 °C. After the incubation, sections were washed in PBS and incubated with horse radish peroxidase (HRP) antibody conjugates (1:500) for 60 min at room temperature. Sections were washed with PBS and the antigen–antibody complexes were visualized using Vectastain Elite ABC Reagent (Vector Laboratories). The sections were developed using DAB (diaminobenzidine) substrate chromogen system (Vector Laboratories) and then counterstained with hematoxylin, dehydrated and mounted with di-n-butylphthalate-polystyrene-xylene (DPX) and the slides were visualized with a Nikon TE300 digital inverted microscope.

### Perls staining

Perls staining was used to detect ferric iron deposits in xenograft sections. The Perls iron staining kit from Abcam (ab150674) was used according to the manufacturer's instructions. Briefly, sections were deparaffinized, hydrated and incubated in equal amounts of potassium ferrocyanide solution and hydrochloric acid solution for 30 min. Slides were washed with water followed by counter staining in nuclear fast red solution for 5 min. After washing, slides were dehydrated, mounted and imaged using a Nikon TE300 digital inverted microscope at 20× magnification. Image J software was used to count the number of Perls positive cells (black pigment) across 3 fields/section were represented graphically.

### Terminal deoxynucleotidyl transferase dUTP nick end labeling (TUNEL) assay

In Situ Detection of Fragmented DNA was examined using an ApopTag peroxidase in situ Apoptosis Detection Kit (S7100) (Millipore, USA) according to the manufacturer’s instructions. The number of TUNEL positive cells in the xenografts were then evaluated using Image J software.

### Bromodeoxyuridine (BrdU) assay

Cells were seeded onto coverslips in 6-well plates at a density of 3 × 10^6^ cells/well and grown overnight prior to incubation with BrdU (3 μg/ml) for 3 h at 37 °C. Cells were fixed with 4% paraformaldehyde for 60 min at room temperature and then washed five times with PBS for 20 min. For immunocytochemical staining, the cells were treated with 0.4% Triton X-100 containing 2 M HCl in PBS for 30 min. After washing with PBS, cells were blocked with 5% horse serum in PBS for 1 h at room temperature and subsequently incubated with mouse monoclonal anti-BrdU antibody (sc-sc-32323, Santa Cruz Biotechnology) at a dilution of 1:200 overnight 4 °C. On the next day, cells were washed in PBS three times and incubated with goat anti-mouse IgG1-Alexa Fluor 488 (A-21121, Invitrogen) at a dilution of 1:100 for 2 h at room temperature in the dark. Following washing three times with PBS each 5 min at room temperature, cells were stained with DAPI (1 μg/ml) for 2–3 min at room temperature to stain nuclei. Fluorescent images were acquired at 20× magnification using a Nikon TE300 digital inverted microscope, and the number of BrdU positive cells were determined by a laboratory member with no knowledge of sample identity using Image J software.

### Kaplan Meier survival analysis

The association between *SNCA* transcript level and melanoma survival was determined using the TCGA (The Cancer Genome Atlas) dataset. To download and obtain the survival data of patients with melanoma, we utilized UCSC Xena Browser (http://xenabrowser.net). UCSC Xena Browser showed that TCGA dataset contained 462 cases of melanoma with both genomic (e.g. *SNCA* mRNA expression) and clinical (e.g. survival rate) information. We then generated a Kaplan Meier survival analysis to analyze the relationship between *SNCA* gene expression and overall survival in patients with melanoma.

### Statistical analyses

Experiments were repeated three times, unless noted otherwise. The statistical significance was determined by a two tailed Student's t-test or a one-way ANOVA with Dunnett posthoc test. All results were expressed as mean ± standard deviation (s.d.) and a statistical difference was accepted at the 5% level unless indicated otherwise. GraphPad prism software or KaleidaGraph was used to make plots.

## Supplementary Information

Supplementary Information.
