# Supplementary material for: Knocking out alpha-synuclein in melanoma cells dysregulates cellular iron metabolism and suppresses tumor growth
Source: Sci Rep. 2021 Mar 4;11:5267. doi: 10.1038/s41598-021-84443-y (PMC7933179; doi:10.1038/s41598-021-84443-y)
Supplement: Supplementary file 1 — Supplementary Information. [file 41598_2021_84443_MOESM1_ESM.pdf]

Supplementary information

Knocking out alpha-synuclein in melanoma cells dysregulates cellular iron metabolism and suppresses tumor growth

Sahar Shekoohi[1], Santhanasababapathy Rajasekaran[1], Dhaval Patel[1], Shu Yang[1], Wang Liu[1], Shile Huang[1], Xiuping Yu[1] and Stephan N. Witt[1,*]

[1]Department of Biochemistry and Molecular Biology, Louisiana State University Health Sciences Center, Shreveport, LA 71103.

*To whom correspondence should be addressed: Tel: 318-675-5163; Fax: 318-675-5180; E-mail: switt1@lsuhsc.edu

Figure S1

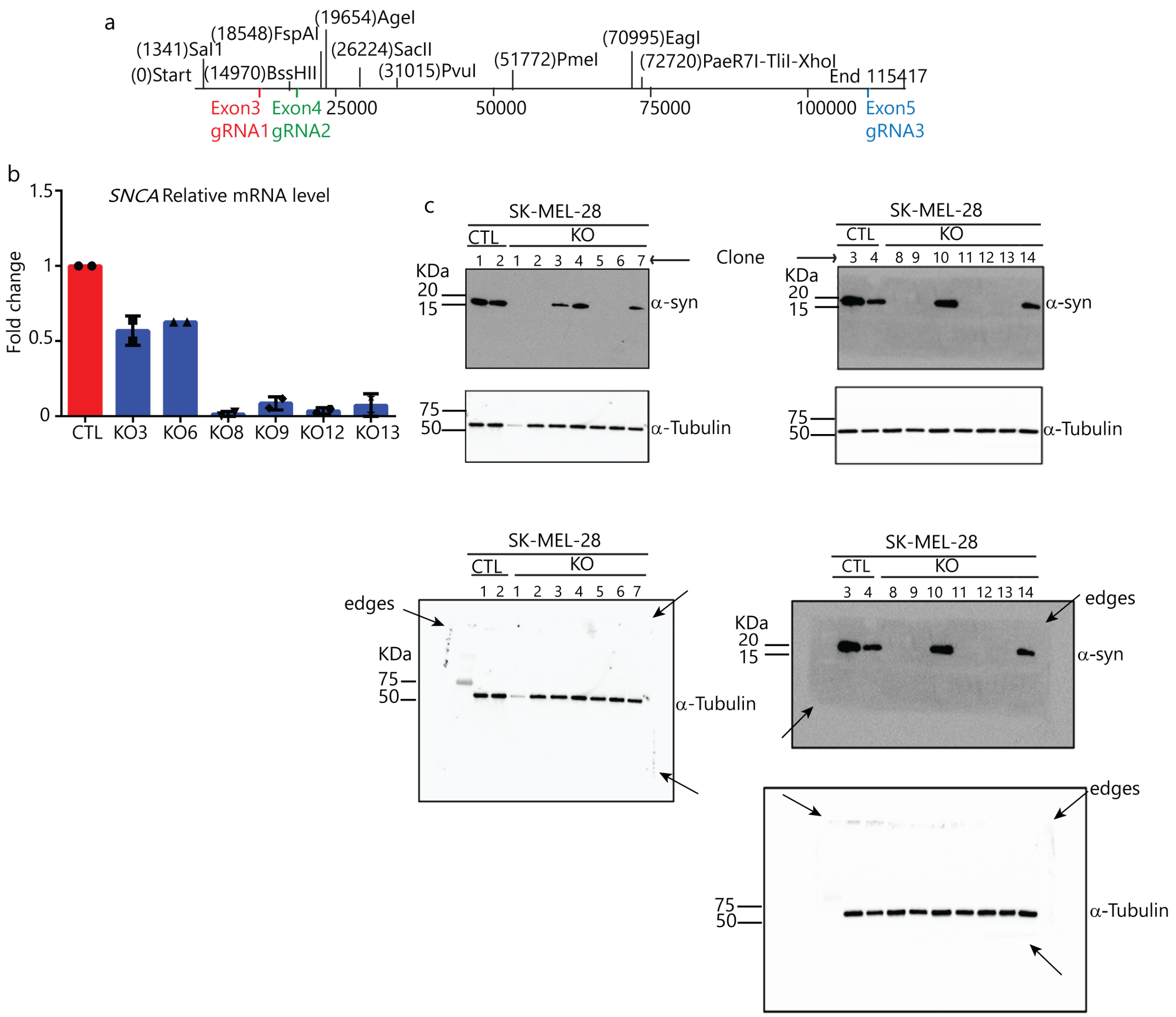

Supplementary Figure S1.

CRISP-Cas9-mediated KO of *SNCA* in SK-Mel-28 melanoma cells. (**a**) Human *SNCA* DNA sequence. gRNA 1, 2 and 3 are complementary to exon 3, 4 and 5, respectively. (**b**) Quantitative data showing RT-qPCR analysis of α-syn transcript level (n=2) in KO cells normalized to control cells and β-actin by ΔΔCt method. (**c**) Western blot analysis of *SNCA* in KO clones (n=3). Cell

lysates were subjected to SDS PAGE followed by western blotting for the indicated proteins. α-Tubulin was used as loading control.

Figure S2

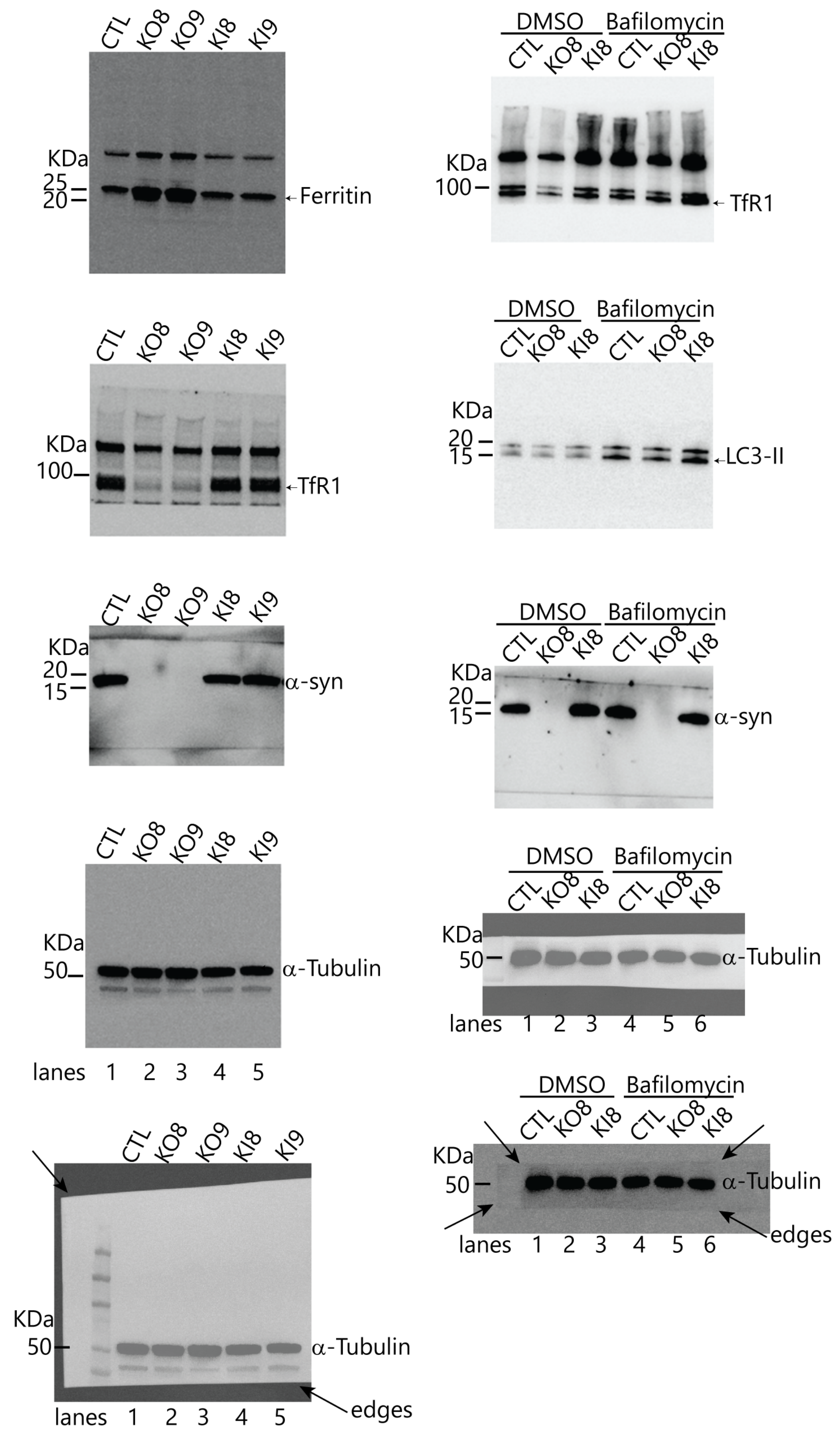
CTL KO8 KO9 KI8 KI9
KDa
25
20
Ferritin
DMSO Bafilomycin
CTL KO8 KI8 CTL KO8 KI8
100
TfR1
LC3-II
15
α-syn
α-Tubulin
50
lanes 1 2 3 4 5
lanes 1 2 3 4 5 6
edges

Figure S2 continued

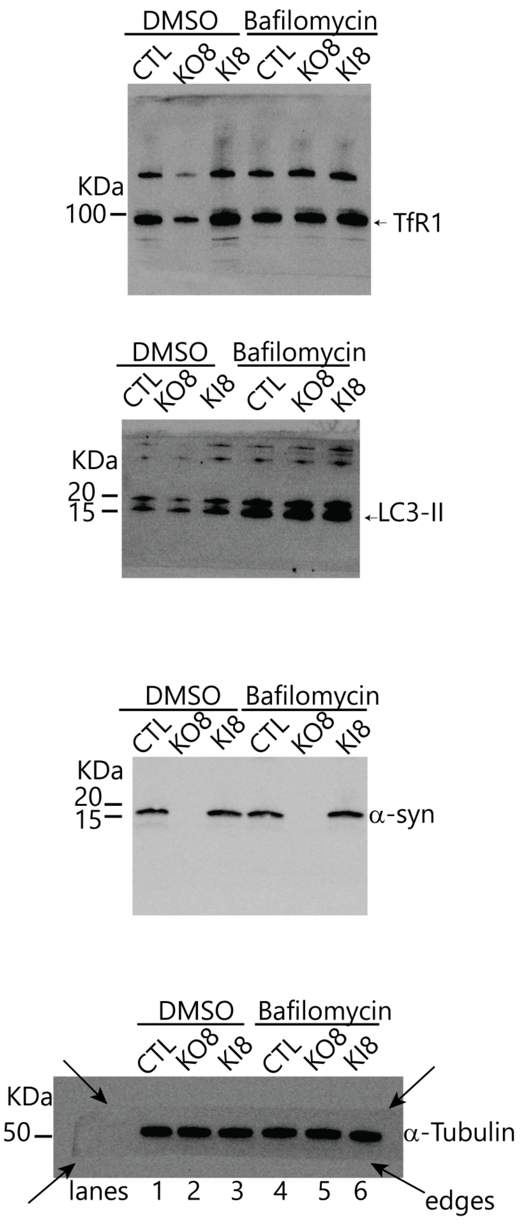

Supplementary Figure S2.

Full length original uncontrasted Western blots which are shown cropped in Figure 2 a, d.

Figure S3

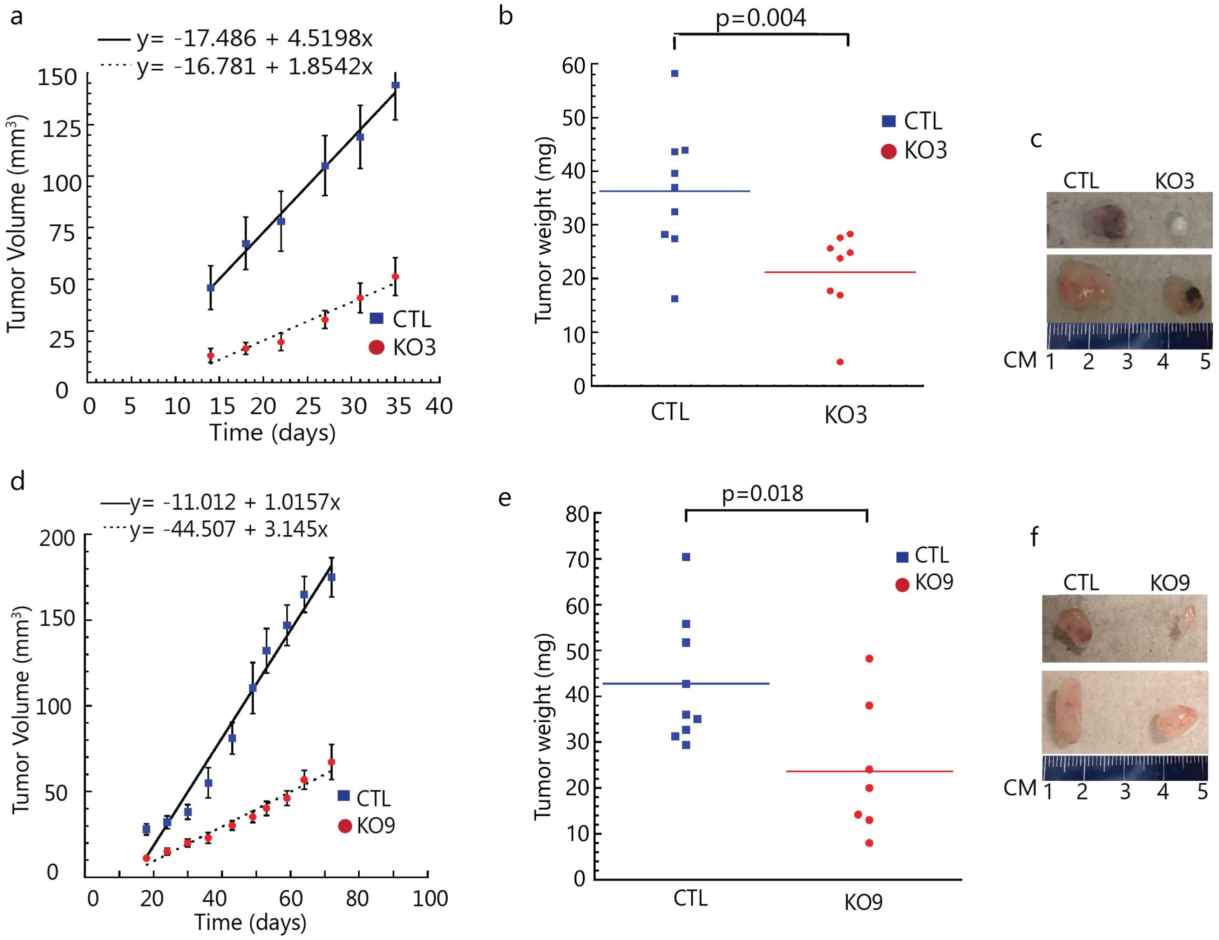

Supplementary Figure S3.

Loss of α-syn expression suppresses cell growth in a mouse xenograft model. (**a**) and (**d**) Tumor volume over time for the control, KO3 and KO9 xenografts. Tumor volume was assessed every 2 days, and average tumor weight was determined after the mice were sacrificed at the end of 72-day experiment. (**b**) and (**e**) Weight of the excised tumors are shown. (**c**) and (**f**) Representative photographs of xenograft mice tumors. Tumor volume and weight were analyzed using two-tailed Student's t test (n = 7-9 mice).

Figure S4

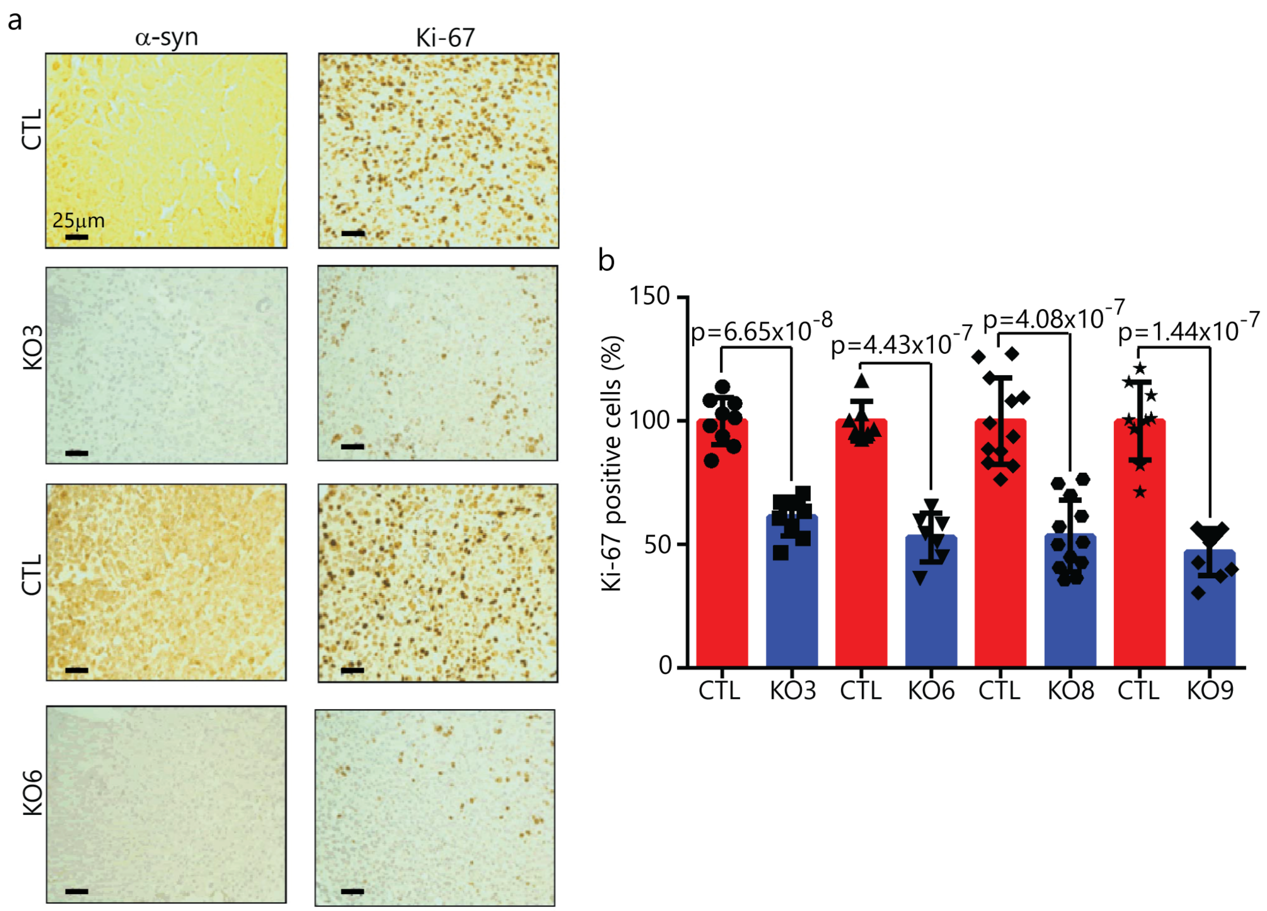

Supplementary Figure S4.

α-syn and Ki67 staining of tumor tissue from SK-Mel-28 xenografts. (**a**) Representative images of immunostained sections of xenografts generated from subcutaneous injection of the *SNCA* KO clone 3, 6 and control cells into mice. Tumor sections were probed with antibodies specific for α-syn and Ki-67. Magnification 20×, scale bar = 25 μm. (**b**) Plot of the number of Ki-67 immunopositive cells per 20x field. For each condition, n = 3 (KO3, 6), with 1-3 fields counted for each slide. The two tailed student's t test was performed to calculate p values. Error bars are ± s.d. This is the same plot as in Figure 6.

Figure S5

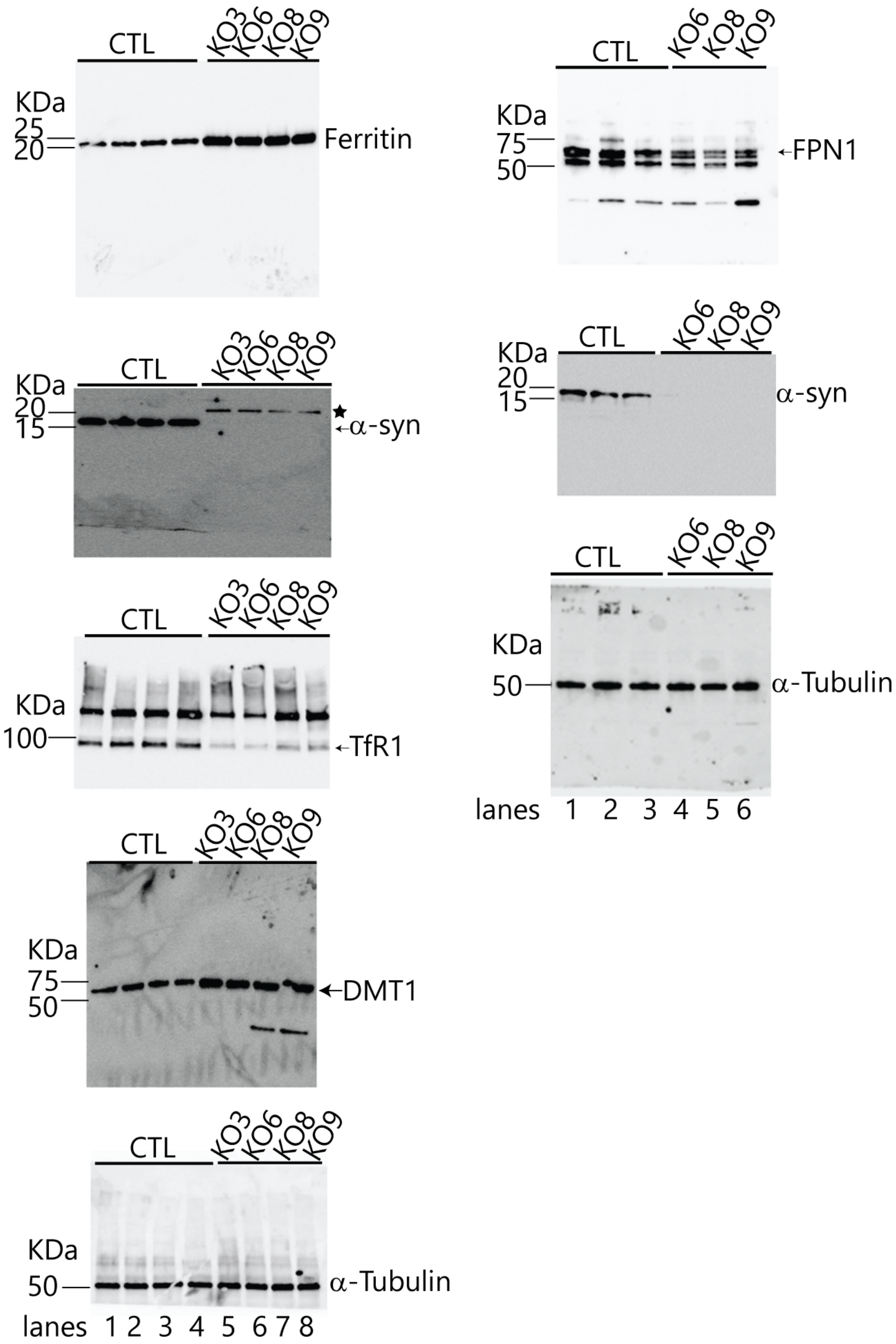

Supplementary Figure S5.

Full length original uncontrasted Western blots which are shown cropped in Figure 7 a, e. *, denotes residual ferritin.

Table S1. SK-Mel-28 *SNCA* KO clones

**Crispr KO clones #**	**WB of α-syn protein**	**RT-qPCR of α-syn mRNA**	**DNA sequencing of *SNCA***	**validation**
Control	yes	yes	All exons	Full expression
3	partial	partial	No exon 4	Monoallelic deletion clone (heterozygous deletion)
6	no	partial	No exon 3; no exon 4	Biallelic deletion clone (homozygous deletion)
8	no	no	No exon 3; no exon 4	Biallelic deletion clone (homozygous deletion)
9	no	no	No exon 3; no exon 4	Biallelic deletion clone (homozygous deletion)

Table S2 Antibodies

Name	Antibody type	Application	Dilution	Catalog Number and Company
α-synuclein	Monoclonal (mouse)	Western Blot / IHC	1:1000/ 1:500	610786, BD Biosciences
Transferrin Receptor	Monoclonal (mouse)	Western Blot	1:1000	13-6800, Thermo Fisher Scientific
Ferroportin	Polyclonal (rabbit)	Western Blot	1:1000	PA522993,Thermo Fisher Scientific
LC3-II	Polyclonal (rabbit)	Western Blot	1:1000	2775S, Cell Signaling Technology
α-Tubulin	Monoclonal (mouse)	Western Blot	1:1000	T9026, Santa Cruz
HRP- conjugated anti-rabbit	rabbit	Western Blot	1:1000	sc-516102, Santa Cruz
HRP- conjugated anti-mouse	mouse	Western Blot	1:1000	sc-2357, Santa Cruz
DMT1	Monoclonal (mouse)	Western Blot	1:1000	sc-166884, Santa Cruz
Ferritin	Monoclonal (mouse)	Western Blot	1:1000	sc-74513, Santa Cruz
Ki-67	Monoclonal (rabbit)	Western Blot / IHC	1:1000/ 1:500	12202S, Cell Signaling Technology
